# Prevalence of Risk Factors for Non-Communicable Diseases in Bangladesh: Results from STEPS Survey 2010

**DOI:** 10.4103/0019-557X.177290

**Published:** 2016

**Authors:** Mohammad Mostafa Zaman, Mujibur Rahman, Ridwanur Rahman, Mahfuzur Rahman Bhuiyan, Nazmul Karim, Abdul Jalil Chowdhury

**Affiliations:** 1 Category Coordinator, World Health Organization, Dhaka, Bangladesh; 2 Professor, Department of Medicine, Shaheed Suhrawardy Medical College, Dhaka, Bangladesh; 3 NCD Risk Factors Survey Physician, Bangladesh Society of Medicine, Dhaka, Bangladesh; 4 National Consultant, NCD Unit, World Health Organization, Dhaka, Bangladesh; 5 Professor, Department of Medicine, Bangabandhu Sheikh Mujib Medical University, Dhaka, Bangladesh

**Keywords:** Bangladesh, epidemiology, noncommunicable diseases (NCDs), population, risk factors, survey

## Abstract

**Background:**

Nationally representative data on noncommunicable disease (NCD) risk factors are lacking in Bangladesh. This study was done to determine the prevalence of common risk factors for major NCDs among men and women of rural and urban areas of Bangladesh.

**Materials and Methods:**

This survey was done with 9,275 individuals aged 25 years or older randomly drawn from all over the country. Information on diet, physical activity, tobacco and alcohol, and treatment history for hypertension and diabetes were collected. Height, weight, waist circumference, and blood pressure (BP) were measured.

**Results:**

There were 4,312 men and 4,963 women with the mean age of 42 years (standard deviation 13 years). Half of them (54%) used tobacco in some form, <1% consumed alcohol within the past 30 days, 92% did not consume adequate fruit and vegetables (five servings or more), and 35% had low physical activity level [<600 metabolic equivalent (MET) min per week]. Documented diabetes was found in 4% of the participants. Seventeen percent were overweight [body mass index (BMI) ≥25 kg/m^2^] and 21% had abdominal obesity (men ≥94, women ≥80 cm). Overall, 21% people had hypertension (blood pressure ≥140/90 mmHg or medication). Physical inactivity, alcohol intake, hypertension, obesity, and diabetes were more prevalent in urban areas, as opposed to tobacco. Tobacco intake showed a decreasing gradient, but hypertension, obesity, diabetes, and low physical activity showed an increasing gradient across the wealth quartiles.

**Conclusion:**

Risk factors are widely prevalent in Bangladeshi people across sexes and across both rural and urban areas of residences. NCD prevention through risk factor control, and early detection and treatment of hypertension and diabetes are warranted.

## Introduction

The current epidemic of noncommunicable diseases (NCDs) is linked to a few common risk factors. These are unhealthy diet, physical inactivity, tobacco, harmful use of alcohol, obesity, raised blood pressure, and raised blood cholesterol and glucose.^1^ The result of these, through a cascade of actions, is to develop major NCDs: Ischemic heart disease, stroke, diabetes mellitus, chronic obstructive pulmonary disease (COPD), and some cancers. These risk factors, fortunately, are largely modifiable or preventable. Therefore, the NCDs are also preventable to a large extent.^2^ Thus, data on risk factors are important for the prediction of future NCDs and also for preventing them at the beginning.

Considering the public health importance of the NCDs and the risk factor approach of NCD prevention, the United Nations General Assembly in its 66th Session in September 2011 unanimously came up with a “Political Declaration of the High-Level Meeting of the General Assembly on the prevention and Control of Non-communicable Diseases.”^3^ As a follow-up action, the World Health Assembly decided to work on nine voluntary targets.^4^ Out of them, six were risk factors: Either to reduce them up to 30% (alcohol, tobacco, insufficient physical activity, high dietary salt intake), or to halt the rising prevalence of diabetes and obesity. Activities to achieve these targets in addition to health system strengthening are expected to bring a 25% relative reduction in the risk of premature mortality from NCDs by 2025.

In Bangladesh, cardiovascular diseases, diabetes, COPD, and cancers have already become a major health problem.^5^ Unfortunately, data on national-level representative risk factors are present only for tobacco,^6^,^7^ and sporadic data are available for other risk factors from small-scale surveys.^8^–^10^ Review of these data indicates a clear, increasing trend of diabetes and hypertension in the Bangladeshi population.^11^

The current survey was done to determine a nationally representative prevalence of tobacco use, alcohol consumption, low fruit and vegetables intake, low physical activity, obesity, hypertension, and diabetes mellitus among men and women in rural and urban areas of Bangladesh.

## Materials and Methods

This survey was conducted as per steps 1 and 2 of the standardized approach devised by the World Health Organization (WHO) known as STEP-wise approach to Surveillance of NCD risk factors (STEPS).^12^ The target population for this survey included all free-living men and women aged 25 years or older (imposing no upper limit of age).

### Sampling strategy and sample size estimation

The sample was drawn from all over the country. The Bangladesh Bureau of Statistics uses the lowest geographical unit, *mauza* in rural areas and *mahalla* in urban areas as their primary sampling units (PSUs). In our study, 400 PSUs (200 *mauza*s and 200 *mahalla*s) were selected randomly with probability proportionate to size, followed by the random selection of households from PSUs. On average, there were 200 households in *mauza*s and 300 in *mahalla*s. People of eligible age who stayed in the household the night before the day of the survey were considered for the survey. Sample substitution was not allowed. Every alternate household was identified as male, and the rest as female households to ensure gender balance.^7^ In the male household, a roster of men was prepared; the same was done for women in the female household to randomly recruit 1 per household using the Kish method.

Using the prevalence of tobacco use of 43.3% within the population^7^ and 3% margin of error, and assuming a design effect of 2, the minimum sample size was 2,096 (rounded to 2,100). This was multiplied by 4 to get a national estimate for four groups (men, women, urban areas of residence, rural areas of residence). Therefore, a minimum of 8,400 respondents was needed. Considering a response of 84%, the sample size was inflated to 10,000 (8,400 ÷ 0.84). Then, to get 10,000 respondents, 25 households per PSU were targeted. Ultimately, 9,275 (93%) individuals could be recruited.

### Survey instruments

An adapted questionnaire for this survey was developed using steps I and II of WHO STEPS.^12^ All the core components of the questions along with some expanded questions were incorporated. The household component of the questionnaire included 20 items of information. The individual component included questions on tobacco, physical activity, alcohol and fruit and vegetable intake. Physical measurements included were height, weight, waist circumference, and blood pressure. Relevant information was also obtained from doctor’s prescriptions, if available on hypertension or diabetes. The questionnaire in Bangla was field-tested before the actual survey. It was entered in a personal data assistant (iPAQ Windows Mobile 5.0 Operating System by Hewlett-Packard Company, Palo Alto, California, USA) for electronic collection and transfer of data to the National Data Center through a secured system of file transfer protocol (FTP) server on a daily basis.

### Data collection

The field team underwent 4-day training before deployment for data collection in the first quarter of 2010. Each of the nine field teams consisted of 1 research physician, and 2 women and 2 men who were professional enumerators. Female respondents were interviewed by female enumerators and male respondents by male enumerators.

### Ascertainment of variables and operational definitions

1. Tobacco: Information on tobacco use was collected for both smoking and smokeless forms. Those who smoked or used smokeless tobacco daily within the past 30 days were considered as “current” users.
2. Alcohol: Alcohol consumption was measured by asking the respondents if they had consumed alcohol within the past 30 days. They were also asked about the frequency of “standard” alcoholic drinking by number of occasions in the past 30 days using pictorial show-cards depicting different types and sizes of glasses used for various alcoholic drinks.^12^
3. Fruit and vegetables: Respondents were asked for the number of days they ate fruit and vegetables in a typical week, and how many servings they ate on one of those days. Servings were measured by showing pictorial show-cards (for uncooked items) or measuring cups (cooked items).
4. Physical activities: Physical activities were measured by asking the respondents about their weekly and daily vigorous and moderate activities during work and leisure time, and during transport, and the time spent in these activities. All types of physical activities were transformed into minutes per day. Metabolic equivalent (MET) min were calculated according to the STEPS protocol as follows: 1 min in moderate and transport-related activities equal to 4 MET min and 1 min in vigorous activities equal to 8 MET min. All MET min for different forms of physical activities were added together to get the total MET min. Then, MET min <600 per week were categorized as low physical activity.
5. Treatment of hypertension and diabetes: Information on treatment of hypertension and diabetes was sought by checking prescriptions or medicine strips if they claimed to be on treatment of these diseases.
6. Anthropometric measurements: Shoes, headgear, footwear, and heavy clothing were removed before measuring height (to the nearest centimeter) and weight (to the nearest 0.2 kg). Waist circumference was measured using a plastic measuring tape midway between the lower margin of the last palpable rib and the top of the hip bone to the nearest 0.5 cm.
7. Blood pressure measurement: Blood pressure was measured using ordinary aneroid sphygmomanometers (in mmHg) on the left arm while the participants were in a sitting position after having a rest for at least 5 min. Korotkoff phase V was taken as diastolic blood pressure. A second reading was taken after 2 min and the mean of these measurements were used in the analysis. Hypertension was defined as blood pressure ≥140/90 mmHg and/or antihypertensive medication.

### Data analysis

Analysis was performed to obtain population prevalence or midpoint estimates and dispersions. We presented mean for quantitative variables with standard deviations with the frequencies of each cell so that any confidence interval can be calculated whenever necessary. In case of skewness, median and interquartile range were presented. For categorical variables, prevalence with frequencies were presented, assuring calculation of confidence interval if needed in future. However, we did not present 95% confidence intervals: our purpose was to describe the distribution of variables. Prevalences were standardized using the new WHO standard population.^13^

In this report, the wealth index of household economic status was created using household assets.^14^ The index was constructed using principal component analysis. The sample was divided in to quartiles from 1 (lowest) to 4 (highest). The distribution of risk factors across the quartiles was then examined. All analyses were done using SPSS version 16.0 (Chicago, Illinois: SPSS Inc.).

### Ethical considerations

Ethical clearance was obtained from het Bangladesh Medical Research Council (BMRC). Just before the interview, written (or thumb impression) consent was obtained from each participant in Bangla as per BMRC guidelines.

## Results

Results are presented in a descriptive manner for age and sex groups separately and combined. Data on key variables for urban and rural residential strata and wealth quartiles are plotted in figures for visual impression of differences. Generally speaking, risk factors are highly prevalent in the Bangladeshi adult population.

### Socioeconomic background

Of the 9275 respondents, 4312 (46.5%) were men [Table 1] with a mean age of 42.4 (±13.5) years. Half of the participants were from urban areas, as stipulated in the study design. Four in 10 of them had completed primary education (men 47% and women 38%) [Table 1]. One-fourth of men were farmers, another fourth were laborers (agriculture, industrial, or otherwise), and one-tenth were salarymen in nonpublic sectors. Of the women, 83% were homemakers.

### Distribution and prevalence of risk factors

Table 2 describes the distribution of risk factors in a quantitative manner, giving midpoints and dispersion, whereas Table 3 describes the prevalence of risk factors in a categorical manner using their cutoff points for normal (or recommended) values.

### Fruit and vegetables

Median per capita consumption of fruit and vegetables was 1.9 servings per day (1.6 servings in men and 2.1 servings in women) [Table 2]. Our finding underlines the suboptimal intake of fruit in Bangladesh population. Although Bangladeshi people eat vegetables almost every day, the amount was found to be low. Only 8.2% of the population [Table 3] consumed the recommended five or more servings of fruit and/or vegetables on an average day.

### Physical activity

More than half of the total physical activity (56%) was contributed by work-related activity, around 31% was contributed by transport-related activity, and the rest was from leisure-time physical activity. Overall median physical activity [Table 2] was 2200 MET min per week (men 5250 MET min per week and women 840 MET min per week). Thirty-five percent of the participants had a low level of physical activity (men 15% and women 54%), taking 600 MET min per week as the cutoff point [Table 3].

### Tobacco

Prevalence of smoking in sexes combined was 27% (men 54.0% and women 1.9%) [Table 2]. They were mainly cigarette and *biri* smokers in isolation or in combination. Age-specific distribution of smoking prevalence was almost homogeneous across age groups in men, but an increasing trend was observed in women. In this survey, 34.2% were smokeless tobacco users. As opposed to smoking, more women (40.4%) were found to use smokeless tobacco than men (29.7%). Older people used smokeless tobacco more than young people. Among the smokeless tobacco users, 66% used *jarda*, 34% used *sada pata*, and 17% used *gul*. When the use of any form of tobacco was considered, 69.9% men and 41.4% women (overall 53.9%) had this bad addition. Among the tobacco users, 20% used both forms of tobacco, smoking or smokeless.

### Alcohol

In this sample, 0.8% (men 1.5% and women 0.1%) were current drinkers (drinking history within past 30 days) [Table 3]. Inversely, 94.4% had never had alcohol in their lifetime. Current drinkers on an average had 5.8 occasions with at least one drink in the past 30 days and consumed on average 3.6 standard drinks on a drinking occasion. Two-third (66.7%) of the current alcohol consumers were binge drinkers (≥5 standard drinks/drinking day for men, ≥4 standard drinks/drinking day for women). On average, current drinkers did binge drinking on 4.2 occasions in the past 30 days.

### Obesity

Mean body mass index (BMI) was 21.5 kg/m^2^ (men 20.9 kg/m^2^ and women 22.0 kg/m^2^) [Table 2]. A substantial proportion (16.9%) of the sample were overweight (BMI ≥25). The prevalence was higher in women (20.2%) than in men (12.8.%) [Table 3]. Average waist circumference (as a measure of central obesity) was 78 cm and 76 cm in men and women, respectively. Overall, more than one in five (21.1%) within the survey population had increased waist circumference (men ≥94 cm, women ≥80 cm). Women (33.0%) had a higher prevalence of central obesity than men (7.9%).

### Blood pressure

One-third of people never measured their blood pressure (BP). Prevalence of self-reported (documented) hypertension was 12.5%. Mean systolic BP was 120 mmHg and diastolic BP was 76 mmHg. Within the survey population, 20.1% (men 19.1% and women 23.0%) had hypertension. The prevalence of hypertension increased with age. Among the people previously reported to have hypertension, only half of them had controlled BP.

### Diabetes mellitus

We determined the prevalence only on the basis of documented evidence of antidiabetic medication. Inquiry revealed that 83% of the survey participants had never had their blood glucose level measured. The prevalence of documented diabetes was 4.4%, with no perceivable sex difference.

### Socioeconomic gradients and clustering of risk factors

**Figure 1:**
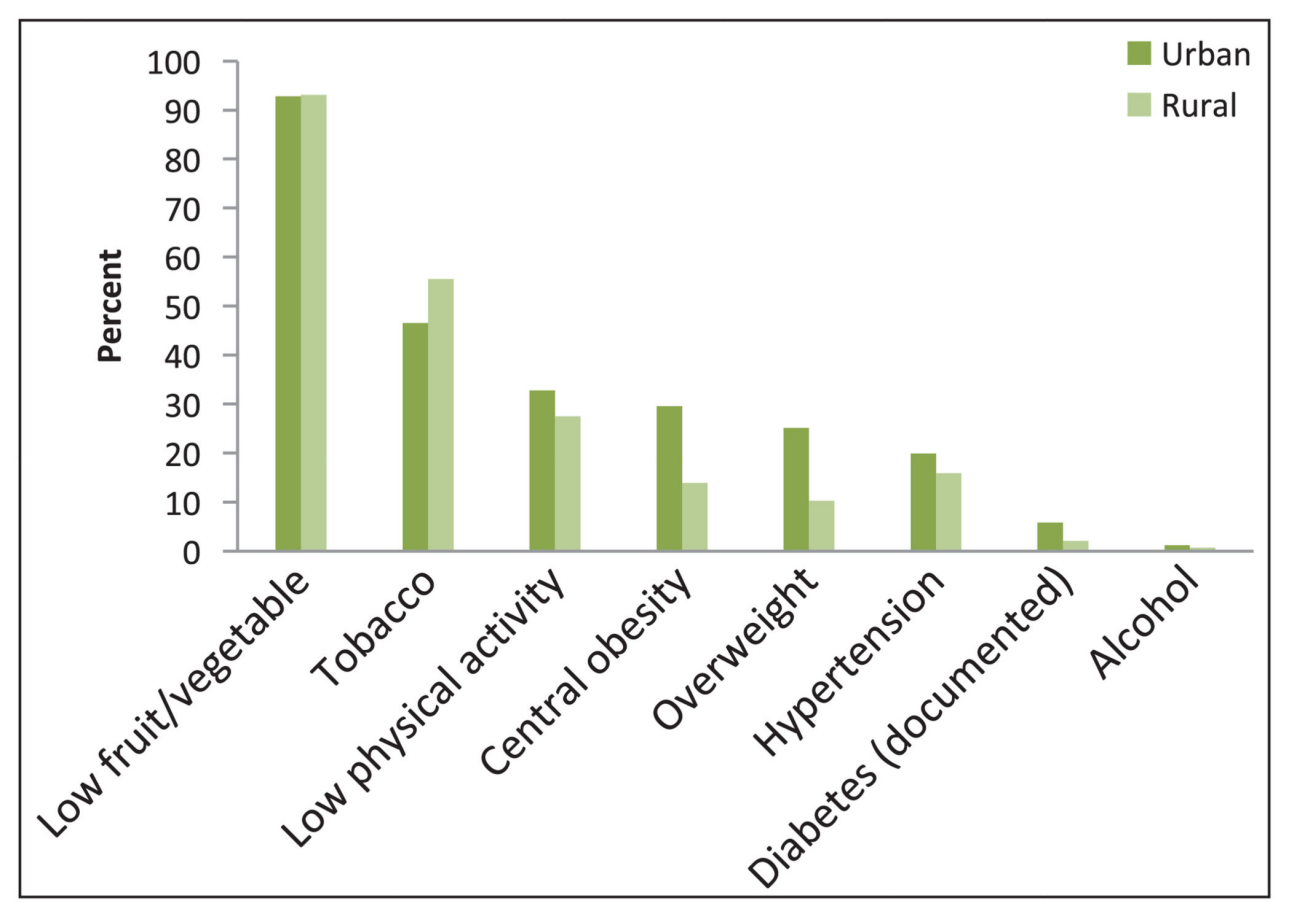
Prevalence (unadjusted) of risk factors stratified into rural and urban areas

**Figure 2:**
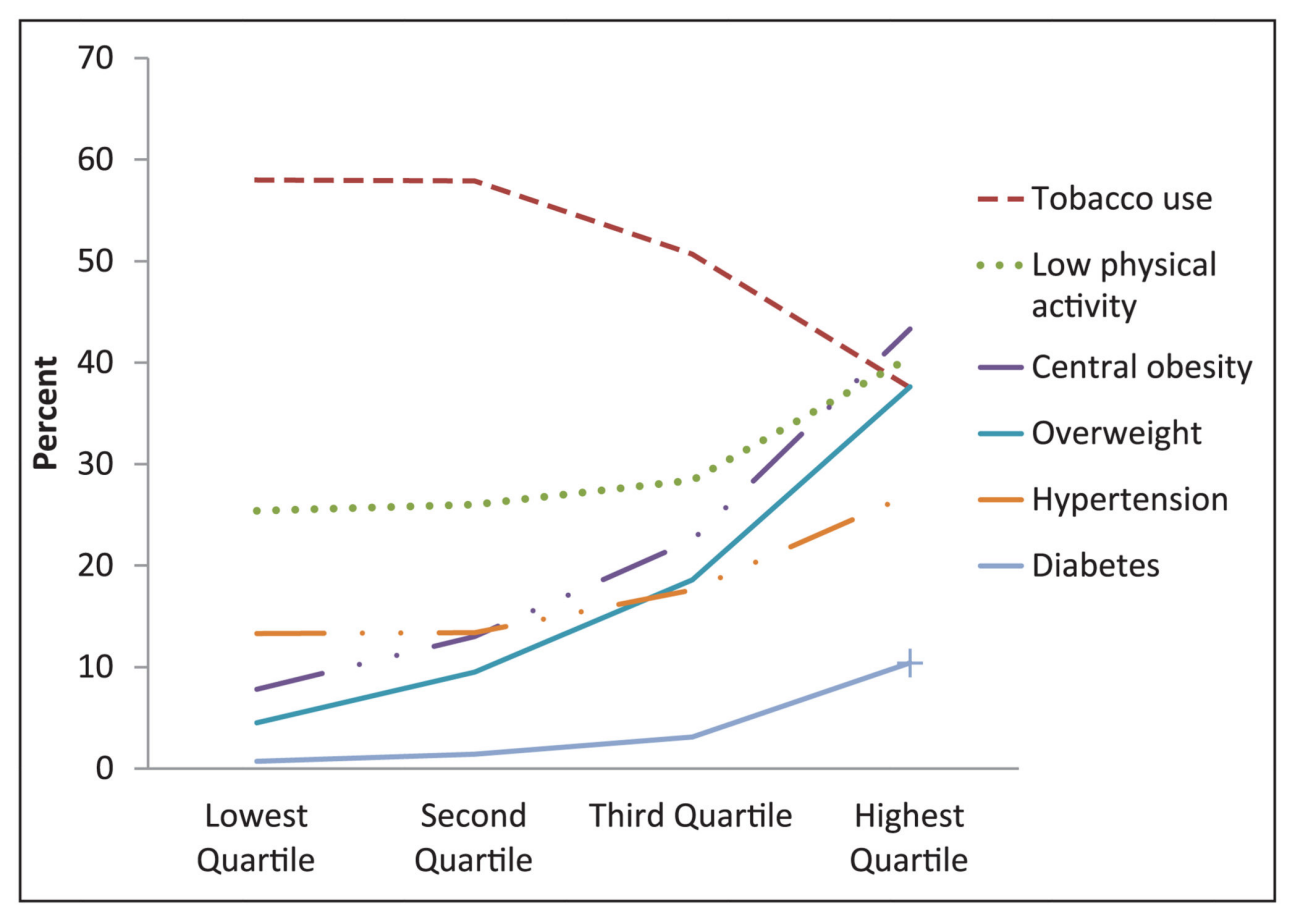
Distribution of risk factors by wealth quartiles

As depicted by Figure 1, low intake of fruit and vegetables was equal in rural urban areas. Tobacco use was the only risk factor to have higher prevalence in rural areas (56% vs 47%). All other factors were more prevalent in urban areas. Alcohol drinking and fruit/vegetable intake remained almost the same across wealth quartiles (not shown in the Figure). Hypertension, low physical activity, obesity, and diabetes prevalence increased but tobacco use decreased with socioeconomic improvement [Figure 2]. As a result, higher proportions of wealthier people are seen to have a clustering of risk factors.

## Discussion

The current study is the second national survey, covering the whole country, on NCD risk factors in Bangladesh. The survey reports a high prevalence of risk factors, which poses a significant threat to the Bangladeshi population for upcoming NCD epidemics. In specific terms, a high prevalence of tobacco use, inadequate fruit and vegetable consumption, and hypertension were observed. The gender and socioeconomic differentials in several factors have also been identified.

Diets low in fruit and vegetables are associated with the increased risk of heart disease, stroke, obesity, and some cancers.^15^,^16^ Strategies aimed at improving dietary habits therefore can play a key role in reducing early deaths from these diseases.^17^ Vegetables are a frequently consumed item in Bangladeshi diet, but the quantity is inadequate. This problem is more prominent in the case of fruit. Local varieties of fruit are sometimes not considered as good fruit by many people. Imported, costly varieties are real fruit to them and considered as a suitable convalescent diet for sick persons. Therefore, a myth reduction strategy has to be in place. There is a need for close cooperation among health and relevant non-health sectors to promote fruit and vegetables issues including production, storage, supply, marketing, fiscal policies, and public awareness for a dietary paradigm shift.

Low physical activity is considered as an important predictor of many chronic NCDs.^18^,^19^ The global estimate for the prevalence of physical inactivity among adults is 17%,^20^ whereas our rate is 33%. Physical inactivity as a problem here cannot be generalized. There are subgroups (such as urban women, a richer segment of the society) that need special attention. Poorly planned urbanization is the major reason for the higher level of physical inactivity in urban areas. Non-health sectors’ (such as local governments, ministry of education, mass transportation, and roads and highways) participation is necessary to promote physical activity. Removal of existing environmental barriers (such as the lack of play grounds, parks, walkable footpaths, or safe roads for bicycles) will play a critical role in promoting physical activity.

Smoking is a major threat to health, given that more than one-fourth of the population are current smokers. A WHO study in 2004 found that 41% of the eight killer diseases (heart attack, stroke, oral cancer, larynx cancer, lung cancer, Buerger’s disease, tuberculosis, and COPD) are attributable to tobacco usage.^4^ The Global Adult Tobacco Survey (GATS) conducted in 2009 reported data for those aged 15 years or older;^5^ ours is for 25 years or older. The prevalence figures in both these studies are almost the same when the analysis is done for the same age group, i.e., 25+ years (data not shown). Using these three sets of data, we suggest that tobacco consumption has reached a plateau in Bangladesh.^11^ Unlike in many other nations, smoking is very low among Bangladeshi women (1.3%). Smokeless tobacco is a common problem in both men and women, and deserves special emphasis. Unfortunately, it was not included in the Smoking Control Act 2005. Tobacco consumption is more prevalent in rural areas compared to urban areas. Use of smokeless tobacco as a component of betel quid has very high cultural acceptance in Bangladesh. Therefore, culturally appropriate campaigning will be required. Considering its public health consequences, the aforesaid Act has already been amended in 2013, incorporating smokeless tobacco.

Obesity has been growing in Bangladesh. The prevalence of overweight in a rural population in 1998 was 6.5%.^21^ It is now 10.2% in rural areas, in the current study [Figure 1], whereas it was 2.5-fold in urban areas. This means that unplanned urbanization has been providing a continuous breeding ground for obesity. Many critics have suggested a lower cutoff point for Asians.^22^ If we apply that BMI>23 kg/m^2^, overall prevalence rises up to 30.4% compared to 16.9% using the conventional one [Table 3]. Central obesity is a special characteristic of the South Asian population in general.^23^ Like general obesity, the central obesity was also more prevalent in women in our sample. These might counter their estrogenic protection (in addition to low smoking rates) for cardiovascular disease development.

Hypertension is the most important risk factor for stroke and heart attacks. We report here a prevalence of 20.8%. Meta-analyses^8^,^24^ and review^11^ of population-based studies on hypertension indicate an increasing trend over the decades, which may reflect a rapid change in lifestyle of the people. Dietary salt intake, the single most important risk factor of hypertension, is very high (11-17 g per day) in Bangladeshi people.^25^ In spite of being a common problem and simple to identify, hypertension detection and treatment status are far from adequate in Bangladesh. Therefore, efficient engagement of the primary health care system needs to be ensured.

We could not measure blood glucose in this study. The actual prevalence of diabetes, we believe, could be at least double what we report here (4.4%) because a prevalence of 6.8% has already been reported in a rural area of Bangladesh.^26^ It is understandable that in urban area it will be even higher.^27^ There are lines of evidences that the prevalence of diabetes is rising in Bangladesh, possibly because of recent, substantial changes in lifestyle.^11^

## Conclusion

Inadequate intake of fruit and vegetables, use of tobacco, low level of physical activity, abdominal obesity, and hypertension are fairly common in Bangladeshi adults. Therefore, a population-based approach using the primary health care system for risk reduction, early detection, and treatment is warranted. Because there is already a large pool of patients with hypertension and diabetes, hospital-based approaches should also be considered. Non-health sectors should be actively engaged for the prevention of risk factors. Control and prevention measures should be designed targeting specific groups of people because there are differences in the distribution of risk factors between rural and urban areas, and based on gender and economic condition. Studies should be undertaken to generate representative data on dietary salt intake, which is still lacking.

## Figures and Tables

**Table 1:** Distribution of the respondents by age, residence and sex

Variables	Men No. (%) (*N* = 4,312)	Women No. (%) (*N* = 4,963)	Both sexes No. (%) (*N* = 9,275)
Age groups			
25-34	1172 (27.2)	1992 (40.1)	3164 (34.1)
35-44	1076 (25.0)	1455 (29.3)	2531 (27.3)
45-54	971 (22.5)	876 (17.7)	1847 (19.9)
55-64	585 (13.6)	430 (8.7)	1015 (10.9)
65+	508 (11.8)	210 (4.2)	718 (7.7)
Residence			
Urban	2175 (50.4)	2454 (49.4)	4629 (49.9)
Rural	2137 (49.5)	2509 (50.5)	4646 (50.1)
Total	4312 (100)	4963 (100)	9275 (100)
Education: Completed primary and above			
All	2044 (47.4)	1880 (37.9)	3924 (42.3)

**Table 2:** Mean (standard deviation) of quantitative risk factors in Bangladeshi adults aged 25 years or older

Age (years)	Number	Fruit/vegetables servings/day*	Physical activity MET-min/week*	Body mass index (kg/m^2^)	Waist circumference (cm)	Systolic blood pressure (mmHg)	Diastolic blood pressure (mmHg)
Men							
25-34	1172	1.6 (1.0, 2.8)	7680 (2805, 17520)	20.8 (3.4)	75.7 (9.2)	116.0 (11.8)	75.1 (9.5)
35-44	1076	1.6 (1.0, 3.0)	5940 (1680, 15600)	21.4 (3.9)	78.6 (10.6)	117.7 (14.6)	77.6 (11.4)
45-54	971	1.6 (1.0, 2.7)	6720 (2520, 16080)	21.3 (5.6)	79.0 (10.5)	122.0 (17.8)	78.9 (11.6)
55-64	585	1.5 (1.0, 2.6)	3400 (1680, 10740)	20.8 (3.8)	78.7 (10.8)	125.9 (21.8)	78.8 (12.7)
65+	508	1.3 (0.8, 2.3)	1920 (370, 4500)	19.7 (3.4)	76.5 (11.9)	133.7 (25.8)	78.6 (13.1)
Total	4312	1.6 (1.0, 2.7)	5250 (1680, 14400)	20.9 (4.2)	77.7 (10.5)	121.2 (18.3)	77.5 (11.5)
Women							
25-34	1992	2.1 (1.2, 3.4)	756 (0, 3360)	22.0 (4.3)	74.9 (10.5)	112.5 (13.2)	72.5 (10.2)
35-44	1455	2.1 (1.3, 3.3)	1140 (80, 4200)	22.3 (4.7)	76.9 (11.7)	118.0 (17.1)	76.4 (11.8)
45-54	876	2.1 (1.2, 3.1)	960 (0, 3360)	21.9 (4.4)	76.3 (12.2)	123.7 (20.0)	78.3 (12.5)
55-64	430	1.9 (1.0, 3.0)	480 (0, 2145)	21.5 (5.1)	75.4 (12.3)	129.9 (23.9)	79.0 (13.5)
65+	210	1.6 (1.0, 2.5)	0 (0, 740)	19.6 (4.2)	71.9 (14.0)	133.9 (24.3)	75.6 (12.4)
Total	4963	2.1 (1.1, 3.2)	840 (0, 3360)	22.0 (4.7)	75.7 (11.6)	118.5 (18.5)	75.3 (11.8)
Both sexes							
25-34	3164	2.0 (1.1, 3.1)	2080 (240, 8160)	21.6 (4.0)	75.2 (10.0)	113.7 (12.8)	73.5 (10.0)
35-44	2531	2.0 (1.1, 3.1)	2400 (480, 8880)	21.9 (4.4)	77.6 (11.3)	117.9 (16.1)	76.9 (11.6)
45-54	1847	2.0 (1.0, 3.0)	3360 (600, 10080)	21.6 (5.4)	77.6 (11.3)	122.8 (18.8)	78.9 (13.0)
55-64	1015	1.6 (1.0, 1.4)	2040 (272, 7200)	21.1 (4.4)	77.3 (11.6)	127.6 (22.8)	78.9 (13.0)
65+	718	1.4 (0.9, 2.4)	960 (0, 3360)	19.7 (3.7)	75.2 (12.7)	133.8 (25.3)	77.7 (13.0)
Total	9275	1.9 (1.0, 3.1)	2200 (320, 8200)	21.5 (4.5)	76.6 (11.1)	119.8 (18.5)	76.3 (11.7)

* Median (interquartile range)

**Table 3:** Prevalence of risk factors in Bangladeshi adults aged 25 years or older [results are number (percent)]

Age (Years)	Number	Fruit/vegetables <5 servings	Low hysical activity**	Smoking	Smokeless tobacco use	Tobacco use*	Drank in past 30 days	Overweight (BMI ≥25 kg/m^2^)	Central obesity***	Hypertension****	Documented diabetes
Men											
25–34	1172	1104 (94.2)	122 (10.4)	638 (54.4)	215 (18.3)	731 (62.4)	32 (2.3)	129 (11.0)	46 (3.9)	74 (6.3)	7 (0.6)
35–44	1076	993 (92.3)	151 (14.0)	648 (60.2)	276 (25.7)	771 (71.7)	26 (1.6)	168 (15.6)	92 (8.6)	142 (13.2)	38 (3.5)
45–54	971	909 (93.6)	100 (10.3)	570 (58.7)	332 (34.2)	726 (74.8)	14 (1.1)	152 (15.7)	103 (10.6)	204 (21.0)	51 (5.3)
55 – 64	585	543 (92.8)	98 (16.8)	313 (53.5)	237 (40.5)	444 (75.9)	8 (1.6)	70 (12.0)	57 (9.7)	182 (31.1)	63 (10.8)
65 +	508	482 (94.9)	167 (32.9)	196 (38.6)	208 (40.9)	348 (68.5)	1 (0.2)	42 (8.3)	46 (9.1)	196 (38.6)	26 (5.1)
Total	4312	4031 (93.5)	638 (14.8)	2365 (54.8)	1268 (29.4)	3020 (70.0)	81 (1.5)	561(13.0)	344 (8.0)	798 (18.5)	185 (4.3)
Age adjusted		93.5	15.4	54.3	29.7	69.9	1.5	12.8	7.9	19.1	4.4
Women											
25-34	1992	1805 (90.6)	1004 (50.4)	8 (0.4)	327 (16.4)	334 (16.8)	1 (0.0)	430 (21.6)	588 (29.5)	128 (6.4)	18 (0.9)
35-44	1455	1354 (93.1)	647 (44.5)	18 (1.2)	531 (36.5)	544 (37.4)	2 (0.1)	346 (23.8)	564 (38.8)	242 (16.6)	55 (3.8)
45-54	876	810 (92.5)	433 (49.4)	15 (1.7)	435 (49.7)	442 (50.5)	2 (0.3)	197 (22.5)	327 (37.3)	237 (27.1)	67 (7.6)
55-64	430	415 (96.5)	256 (59.5)	10 (2.3)	245 (57.0)	251 (58.4)	1 (0.2)	82 (19.1)	146 (34.0)	153 (35.6)	31 (7.2)
65 +	210	206 (98.1)	157 (74.8)	13 (6.2)	132 (62.9)	138 (65.7)	0 (0)	19 (9.0)	47 (22.4)	99 (47.1)	9 (4.3)
Total	4963	4590 (92.5)	2497 (50.3)	64 (1.3)	1670 (33.6)	1709 (34.4)	6 (0.1)	1074 (21.6)	1672 (33.7)	859 (17.3)	180 (3.6)
Age adjusted		93.5	53.5	1.9	40.4	41.4	0.1	20.2	33.0	23.0	4.3
Both sexes											
25-34	3164	2909 (91.9)	1126 (35.6)	646 (20.4)	542 (17.1)	1065 (33.7)	33 (0.9)	559 (17.7)	634 (20.0)	202 (6.4)	25 (0.8)
35-44	2531	2347 (92.7)	798 (31.5)	666 (26.3)	807 (31.9)	1315 (52.0)	28 (0.8)	514 (20.3)	656 (25.9)	384 (15.2)	93 (3.7)
45-54	1847	1719 (93.1)	533 (28.9)	585 (31.7)	767 (41.5)	1168 (63.2)	16 (0.7)	349 (18.9)	430 (23.3)	441 (23.9)	118 (6.4)
55-64	1015	958 (94.4)	354 (34.9)	323 (31.8)	482 (47.5)	695 (68.5)	9 (1.0)	152 (15.0)	203 (20.0)	335 (33.0)	94 (9.3)
65+	718	688 (95.8)	324 (45.1)	209 (29.1)	340 (47.4)	486 (67.7)	1 (0.2)	61 (8.5)	93 (13.0)	295 (41.1)	35 (4.9)
Total	9275	8621 (92.9)	3135 (33.8)	2429 (26.2)	2938 (31.7)	4729 (51.0)	87 (0.9)	1635 (17.6)	2016 (21.7)	1657 (17.9)	365 (3.9)
Age adjusted		91.8	34.5	27.0	34.2	53.9	0.8	16.9	21.1	20.8	4.4

* Daily or nondaily tobacco users of any tobacco product, smoking or smokeless

** Less than 600 MET-min per week

*** Waist circumference of men ≥94 cm and women ≥80 cm

**** Blood pressure ≥140/90 mmHg and/or medication
